# Prevalence of Migraine among patients of Depressive Disorder

**DOI:** 10.12669/pjms.344.14693

**Published:** 2018

**Authors:** Muhammad Ilyas Jat, Muhammad Iqbal Afridi, Washdev Amar, Chooni Lal

**Affiliations:** 1 Dr. Muhammad IlyasJat, FCPS. Department of Medicine and Allied, Isra University, Karachi Campus, Al-Tibri Medical College, Malir, Karachi, Pakistan; 2 Dr. Muhammad Iqbal Afridi, FCPS, FRCP. Department of Psychiatry and Behavioral Sciences, Jinnah Postgraduate Medical Centre, Karachi, Pakistan; 3 Dr. Washdev, FCPS. Department of Psychiatry, Dow University of Health Sciences, Karachi, Pakistan; 4 Dr. Chooni Lal, FCPS. Department of Psychiatry and Behavioral Sciences, Jinnah Postgraduate Medical Centre, Karachi, Pakistan

**Keywords:** Migraine, Depressive disorder, Prevalence

## Abstract

**Objective:**

To find out the prevalence of Migraine among patients of Depressive Disorder.

**Methods:**

A descriptive cross sectional study, conducted at Department of Psychiatry and Behavioral Sciences, JPMC, Karachi from 1^st^ January 2014 to 30^th^ June 2014. Total 272 patients were enrolled in the study. Depressive disorder was diagnosed as per ICD-10 criteria and Migraine headache as ICHD-2 criteria for diagnosis.

**Results:**

A total of 272 patients with mean age of 31.85 ± 8.7 were enrolled. Out of 272 cases 64% were females; Out of total cases 86.4% were married. Migraine with aura was seen among 6.6% and migraine without aura was present among 26.1%. Migraine was linked more with females and married and of those having severe Depressive disorder.

**Conclusion:**

Migraine headache is common among depressed people, particularly females and having severe depression, so it ought to be remembered that while looking for Depressive disorder or headache the other condition must be remembered.

## INTRODUCTION

Depression/Depressive disorder is a mood disorder, characterized by low mood, lack of interest and enjoyment, reduced self-esteem, slowness and reduced energy, disturbed sleep and appetite, leading to decreased social and occupational functioning. Life time prevalence of Depressive disorder is 18 to 22% and one year rate is 2-5% having greater ratio in females than males.^1^ Migraine is a chronic neurological headache disorder accompanied with some autonomic nervous symptoms such as nausea, vomiting, photophobia (sensitivity to light) and phonophobia (sensitivity to sound). The International Classification of Headache Disorders (ICHD) published by the International Headache Society (IHS)^2^ is used as the most reliable and acceptable diagnostic criteria for migraine. According to the IHS criteria, previous studies consistently showed a lifetime prevalence of migraine at 10–20%.^3^ Both migraine and depression are common complex diseases with complicated inheritance patterns, episodic manifestations and great burdens on general populations.^4^ The increased chances of having depression while suffering from migraine is an example of an association known in epidemiological jargon as “bi-directional co-morbidity”.^5^ It is pertinent to note that the classification of migraine can be divided into six major types of which the “migraine with aura” sub-type has the highest association with the risk of developing depression.^6^ The bi-directional relationship between these two disease entities has been scrutinized and confirmed by in-depth and contemporary studies.^7^-^10^ The above mentioned studies highlight a need to further explore this strong association of depression and migraine: Thus, this study is designed to reveal the burden of both illnesses simultaneously.

## METHODS

This is a descriptive cross sectional type of study and was conducted at Department of Psychiatry and Behavioral Sciences, Jinnah Postgraduate Medical Centre (JPMC), Karachi, Pakistan from 1^st^ January 2014 to 30^th^ June 2014.The sample size of 272 cases was calculated through standard sample size calculation. Ethical considerations were taken and prior ethical approval from institute was taken, informed consent was sought from every client. Both male and females patients diagnosed with depressive disorder were enrolled. Patients, who were substance users, had Depressive Disorder due to another medical condition or due to any drug patient is taking, patients of Bipolar Depression, Post- Schizophrenic Depression or Depressive Disorder with Psychotic features were excluded from study.

All depressive disorders (based on ICD-10 criteria) patients were seen for Migraine over the diagnostic criteria for migraine from International Classification of Headache Disorders second edition (ICHD-2). The data was analyzed on SPSS version 20.

## RESULTS

A total of 272 patients with mean age of 31.85 ± 8.7 were enrolled. Out of 272 cases 174(64%) were females and 98(36%) were males. Out of total cases 235(86.4%) were married and 36(13.20%) were single and 1(0.4%) was widow. Out of total 86 (31.6%) were uneducated, 50(18.40%) were literate, 13(4.8%) were primary passed, 29(10.7%) were educated till middle and 34(12.5%) were matriculated and 54(19.9%) were intermediate and 6(2.2%) were graduated. Among all clients 74(27.2%) were jobless, 174(64%) were household, 13(4.8%) were students and 7(2.6%) were professionals as shown in Table-II demographic characteristics. Migraine with aura was seen among 18(6.6%) and migraine without aura was present among 71(26.1%) shown in Table-III prevalence of Migraine. Migraine was associated more with female gender and those of married and of those having severe Depressive disorder. Stratification of migraine found to be significant with gender as more common among females and with occupation as more prevalent among household workers.

**Table-I:** Frequency of Migraine.

Migraine	Frequency	%
No Migraine	183	67.3
Migraine with aura	18	6.6
Migraine without aura	71	26.1

Total	272	100

**Table-II:** Association of Migraine with Depressive disorder.

Migraine	Depressive Disorder		Total	P-Value

	Moderate	Severe		
No Migraine	47(25.7%)	136(743%)	183(100%)	0.566
Migraine with aura	3(16.7%)	15(83.3%)	18(100%)	
Migraine without aura	15(21.1%)	56(78.9%)	71(100%)	

Total	65(23.9%)	207(76.1%)	272(100%)	

**Table-III:** Association of Migraine with gender.

Migraine	Gender		Total	P-Value

	Male	Female		
No Migraine	88(48.1%)	95(51.9%)	183(100%)	0.000
Migraine with aura	1(5.6%)	17(94.4%)	18(100%)	
Migraine without aura	9(12.7%)	62(87.3%)	71(100%)	

Total	98(36%)	174(64%)	272(100%)	

**Table-IV:** Association of Migraine with Occupational and Educational Status.

Migraine	Occupation							Total	P-Value

	Student	House-hold		Professional	Shopkeeper		Jobless		
No Migraine	07(3.8%)	96(52.5%)		06(3.3%)	04(2.2%)		70(38.3%)	183(100%)	0.000
Migraine with aura	00(0%)	17(94.4%)		00(0%)	00(0%)		01(5.6%)	18(100%)	
Migraine without aura	06(8.5%)	61(85.9%)		01(1.4%)	00(0%)		03(4.2%)	71(100%)	

Total	13(4.8%)	174(64%)		07(2.6%)	04(1.5%)		74(27.2%)	272(100%)

**Migraine**	**Educational Status**							**Total**	**P-Value**

	**Uneducated**	**Literate**	**Primary**	**Middle**	**Matric**	**Intermediate**	**Graduate**		

No Migraine	61(33.3%)	28(15.3%)	08(4.4%)	21(11.5%)	22(12.0%)	38(20.8%)	05(2.7%)	183(100%)	0.348
Migraine with aura	07(38.9%)	06(33.3%)	02(11.1%)	00(0%)	00(0%)	03(16.7%)	00(0%)	18(100%)	
Migraine without aura	18(25.4%)	16(22.5%)	03(4.2%)	08(11.3%)	12(16.9%)	13(18.3%)	01(1.4%)	71(100%)	

Total	86(31.6%)	50(18.4%)	13(4.8%)	29(10.7)	34(12.5%)	54(19.9%)	06(2.2%)	272(100%)

## DISCUSSION

Important findings of this study are that Migraine is prevalent in depressive disorder and Migraine without aura is predominant. Migraine in this study is 26.1% which is higher as compared to previous study conducted in India during 2006 and 2007, in which Migraine was found to be 12.5%.^11^ While in another study conducted by Naila et al found Migraine with depressive disorder was in 40% of the study patients.^12^ We have found out the prevalence of Migraine among depressive disorder patients as 26.1% which is quite low as compared to a study in which they observed it was 44.44%,^13^ selected on the basis of International Headache Society (HIS) criteria for migraine while we have used International Criteria for Headache Disorders second edition (ICHD-2) for Migraine diagnosis and in that study depression was based on 21-item HRDS for depression while we diagnosed depression as per ICD-10 criteria. In a population-based case control study, Lipton et al found that 47% of migraine patients developed depressive disorder, as compared to 17% of people who do not have migraine^14^; here we have seen Migraine among depressed patients rather than depression among migraine sufferers. A study conducted at Karachi, Pakistan in 2013 showed the results consistent with our results, in that research Migraine was 20.0% as compared with ours 26.1%.^15^

In this study, Migraine headache is more associated with female gender as evidenced in a study conducted at multiple centers across Pakistan.^16^ The important finding of this study is that Migraine is found more prevalent among females and those of household and this has not been looked so far. However, almost the majority of the evidence on the relationship between Migraine headache and depressive disorder is from studies on group samples or center based samples of clients having headache,^17^ as opposed to individuals experiencing depressive disorder. Here in our study, we have embarked to analyze the quality and specificity of the relationship amongst depressive disorder and migraine on an expansive example of individuals experiencing depressive disorder.

## CONCLUSION

Migraine headache is common among peoples of depressive disorder, particularly females and having severe depression so it should to be remembered that while looking for Depressive disorder or headache the other condition ought to be remembered. Depression, when it is co-morbid with the migraine, not just increases the duration, recurrence and severity of disorder, yet in addition makes it more impervious to treatment. It likewise creates a more profound effect on personal satisfaction for the influenced individual and general expands the weight of the illness.

### Author’s Contribution

**MIJ:** Data collection, writing of manuscript, statistical analysis.

**MIA:** Did review and final approval of manuscript.

**WA:** Editing of manuscript, Statistical analysis.

**CL:** Data collection and writing of manuscript.

## References

[1] Gelder M, Paul H, Cowen P (2006). Mood Disorders. Shorter Oxford text book of psychiatry.

[2] (2013). Headache Classification Committee of the International Headache S. The International Classification of Headache Disorders.

[3] Arroyo-Quiroz C, Kurth T, Cantu-Brito C, Lopez-Ridaura R, Romieu I, Lajous M (2014). Lifetime prevalence and under diagnosis of migraine in a population sample of Mexican women. Cephalalgia.

[4] Vos T, Flaxman AD, Naghavi M, Lozano R, Michaud C, Ezzati M (2012). Years lived with disability (YLDs) for 1160 sequelae of 289 diseases and injuries 1990-2010:a systematic analysis for the Global Burden of Disease Study 2010. Lancet.

[5] Amoozegar F (2017). Depression comorbidity in migraine. Int Rev Psychiatry.

[6] Serrano-Duenas M (2000). Chronic tension-type headache and depression. Rev Neurol.

[7] Yang Y, Ligthart L, Terwindt GM (2016). Genetic epidemiology of migraine and depression. Cephalalgia.

[8] Risal A, Manandhar K, Holen A, Steiner TJ, Linde M (2016). Comorbidities of psychiatric and headache disorders in Nepal:implications from a nationwide population-based study. J Headache Pain.

[9] Minen MT, De Dahem OB, Van Diest AK, Power S, Schwedt TJ, Lipton R (2016). Migraine and its psychiatric comorbidities. J Neurol Neurosurg Psychiatry.

[10] Goulart AC, Santos IS, Brunoni AR, Nunes MA, Passos VM, Griep RH (2014). Migraine headaches and mood/anxiety disorders in the ELSA Brazil. Headache.

[11] Gupta R, Bhatia MS, Singh NP, Upreti R (2007). Association of Depression with Headache. J Pak Psy Soc.

[12] Shehbaz N, Ali S, Akhtar SW, Aziz H (2007). Migraine:Comorbidity with depression. Pak J Med Sci.

[13] Juang KD, Wang SJ, Lin CH, Fuh JL (1999). Use of the hospital anxiety and depression scale as a screening tool for patients with headache. Zhonghua Yi Xue Za Zhi (Taipei).

[14] Lipton RB, Hamel sky SW, Kolodner KB, Steiner TJ, Stewart WF (2000). Migraine, quality of life, and depression:a population-based case-control study. Neurology.

[15] Arif D, Akbar A, Ali A, Umer L, Bilal A, Jahanzeb E (2013). The burden of headache disorders in Pakistan:methodology of a population-based nationwide study, and questionnaire validation. J Headache Pain.

[16] Herekar AA, Ahmad A, Uqaili UL, Ahmed B, Effendi J, Alvi SZ (2017). Primary headache disorders in the adult general population of Pakistan –a cross sectional nationwide prevalence survey. J Headache Pain.

[17] Radat F, Swendsen J (2005). Psychiatric comorbidity in migraine:a review. Cephalalgia.

